# The Associations between Anthropometric Indices and Obstructive Sleep Apnea in a Korean Population

**DOI:** 10.1371/journal.pone.0114463

**Published:** 2014-12-04

**Authors:** Hyeon Hui Kang, Ji Young Kang, Jick Hwan Ha, Jongmin Lee, Sung Kyoung Kim, Hwa Sik Moon, Sang Haak Lee

**Affiliations:** Division of Pulmonary, Critical Care and Sleep Medicine, Department of Internal Medicine, College of Medicine, The Catholic University of Korea, Seoul, Republic of Korea; University Medical Center Groningen UMCG, Netherlands

## Abstract

**Background:**

Obesity is a major risk factor for the development of obstructive sleep apnea (OSA). Although clinical and epidemiological studies have shown that OSA and obesity are strongly associated, few Asian studies have examined the associations between anthropometric obesity indices and OSA, especially in the Korean population. The purpose of this study was to evaluate the influence of anthropometric obesity indices on OSA in a Korean population.

**Methods:**

Anthropometric indices, including neck circumference, waist circumference, and body mass index, were assessed in 383 consecutive subjects with suspected OSA.

**Results:**

Of the 383 subjects assessed, 316 (82.5%) were diagnosed with OSA. Neck circumference (r = 0.518), waist circumference (r = 0.570), and body mass index (r = 0.512) were correlated with the apnea-hypopnea index (p<0.001, for all). After adjusting for age, sex, alcohol consumption, and smoking, a logistic regression model showed that neck circumference [odds ratio (OR), 1.414; p<0.001)], waist circumference (OR, 1.114; p<0.001), and body mass index (OR, 1.364; p<0.001) were associated with OSA. The linear regression model showed that neck circumference (β = 3.748, p<0.001), waist circumference (β = 1.272, p<0.001), and body mass index (β = 3.082, p<0.001) were associated with apnea-hypopnea index. The cut-off values for predicting OSA were determined as 34.5 cm for neck circumference, 76.5 cm for waist circumference, and 23.05 kg/m^2^ for body mass index for females, and 38.75 cm for neck circumference, 88.5 cm for waist circumference, and 24.95 kg/m^2^ for body mass index for males.

**Conclusion:**

Increased anthropometric indices were significantly associated with the presence and severity of OSA in a Korean population. In addition, this study demonstrated the cut-off values for body mass index, waist circumference, and neck circumference for increased OSA risk.

## Introduction

Obstructive sleep apnea (OSA), which is the most severe form of obstructive sleep-disordered breathing (SDB), is characterized by repeated collapse of the upper airway due to a marked reduction or complete cessation of airflow during sleep [1]. Many studies have reported that obesity, particularly central obesity, is significantly associated with an increased prevalence of OSA in the general population, because it promotes enlargement of the soft tissue structures within and surrounding the airway, thereby contributing significantly to pharyngeal airway narrowing [2]. The association between OSA and obesity is complex. Despite the preponderance of evidence linking obesity and OSA, appreciable variability exists in the prevalence and severity of OSA. In severely obese patients who underwent bariatric surgery, the severity of OSA did not correlate with the degree of obesity, as assessed by body mass index (BMI) [3]. Body fat distribution also seems to be of importance in the relationship between OSA and adiposity as well as weight gain [4]. Clinically, neck circumference (NC) has been known to be a strong predictor of OSA [5], [6]. However, some authors have demonstrated that BMI correlates with OSA better than NC, especially in women with OSA [7]. However, the criteria of obesity are dependent on sex and ethnicity [8], and the relationship between obesity and OSA is unclear.

Although polysomnograpy is an essential tool for diagnosis of OSA, it is costly, complex, and, in many countries, difficult to access [9]. Therefore, determining patients who are at a high risk patient for is very important when considering the use of full overnight polysomnography to diagnosis OSA. Obesity measures for predicting of OSA may be useful, and it is important for sleep physicians to know the optimal cut-off values of the anthropometric obesity indices and their diagnostic significance. Although many epidemiological studies have shown a relationship between OSA and anthropometric indices, only the mean values of the anthropometric indices that were associated with OSA have been reported, and few studies have reported the optimal cut-off values for BMI, NC, and waist circumference (WC) in patients with OSA [10], [11], especially those in the Asian population. This study aimed to evaluate BMI, NC, and WC in patients with and without OSA and determined the cut-off points for the anthropometric obesity indices that were related to the presence and severity of OSA.

## Materials and Methods

### Ethics statement

This study was approved by institutional review board of St. Paul's Hospital (IRB approval number: PC14OISI0059). The institutional review board approved the protocol, and all participants gave written informed consent.

### Subjects and study design

We analyzed the data for 383 consecutive patients who were admitted to the sleep clinic at St. Paul's Hospital, College of Medicine, The Catholic University of Korea for evaluation of OSA between 2007 and 2012. All of the patients had at least one of the symptoms, including snoring, witnessed apneic incidents, excessive daytime sleepiness, and nocturnal gasping or choking, and were suspected of having OSA.

An overnight polysomnographic evaluation with a Somnostar Pro 7-3a (Cardinal Health, Inc., Dublin, OH, USA) was performed in all of the patients. The procedure was composed of polygraphic recordings from surface electrodes for electroencephalography, electrooculography, electrocardiography, and electromyography and from nasal pressure transducer with thermistors for nasal and oral airflow, tracheal sounds, and thoracic and abdominal respiration. The monitoring of transcutaneous oxygen saturation was performed continuously with a finger pulse oximeter. Positional changes during sleep were recorded, and the full-night video recordings were also performed during the test period. All of the evaluations were terminated after the final waking in the morning. The data were collected in a computerized polysomnographic system, and the scoring process was performed manually. Sleep was defined according to the criteria of Rechtschaffen and Kales [12]. Respiratory events were scored according to the criteria of the American Academy of Sleep Medicine [13]. Apnea was defined as a cessation of airflow lasting at least 10 s. The hypopnea was defined if there was a reduction of airflow measured by nasal pressure transducer ≥30% that lasted ≥10 s along with ≥4% oxygen desaturation, or 50% or more decrease in airflow lasting at least 10 s, a discernible decrease of 3% or more oxygen saturation, or an electroencephalography arousal. The apnea-hypopnea index (AHI) was defined as the number of apnea and hypopnea events that occurred per h of sleep. The AHI, when associated with typical symptoms, was scored as follows: AHI ≥5 events/h was diagnosed as OSA, of these, 15≥AHI≥5 events/h was considered mild, 30≥AHI>15 events/h was considered moderate, and>30 events/h was considered severe OSA [14]. Those who had an AHI less than 5 were included in the non-OSA group.

The BMI was calculated as weight (kg) ÷ height (m^2^). NC was measured at the level of the cricothyroid membrane, and WC was measured at the midpoint between the lower border of the rib cage and the iliac crest in the upright position. Patients with Epworth sleepiness score 10 or more were considered to have excessive daytime sleepiness [15].

### Statistical analysis

The data were expressed as mean ± standard deviation for continuous variables and as numbers and percentages, n (%), for categorical variables. Continuous variables were compared with Student's *t*-tests, and categorical variables were compared with a χ^2^ test or Fisher's exact test, as appropriate. The correlations between the variables were assessed with a Pearson's correlation test. After adjusting for age, sex, alcohol consumption, and smoking status, a logistic regression analysis was performed to evaluate the significance of the individual anthropometric indices for the presence of OSA. A linear regression analysis was performed to evaluate the significance of the individual anthropometric indices for the severity of OSA. A receiver operating characteristic (ROC) curve analysis with Youden index was performed to determine the optimal cut-off point for the individual anthropometric indices including BMI, NC, and WC, for predicting OSA, in both males and females. SPSS version 18 (Chicago, Illinois, USA) was used for all of the statistical analyses. P-values less than 0.05 were considered statistically significant.

## Results

The clinical characteristics of the 383 patients (age, 48.4±14 years; 72% men) in this study are presented in Table 1. This study included 316 patients with OSA (82.5%) and 242 males with OSA (77%).

**Table 1:** Baseline characteristics of the study subjects.

	Non-OSA (n = 67)	OSA (n = 316)	*P value*
Age (years)	44±17	48.4±13	0.012
Male, n (%)	33 (49)	242 (77)	<0.001
Smoking, n (%)	16 (24)	128 (41)	0.012
Alcohol consumption, n (%)	37 (55)	201 (64)	0.212
Body mass index (kg/m^2^)	23.1±2.9	26.8±3.9	<0.001
Waist circumference (cm)	83.5±9.0	94.5±10.2	<0.001
Neck circumference (cm)	34.4±3.2	38.1±3.6	<0.001
Epworth sleepiness scale	8.2±3.7	9.4±4.2	0.040
Mean SaO_2_ (%)	95.9±1.5	92.1±4.0	<0.001
Lowest SaO_2_ (%)	90.2±3.1	79.2±8.5	<0.001
Hypopnea index (/h)	1.3±1.2	13.8±11.6	<0.001
Apnea index (/h)	0.9±1.1	22.0±23.2	<0.001
Apnea-hypopnea index (/h)	2.1±1.5	35.8±26.2	<0.001
Sleep period time (min)	419.2±48.2	408.9±49.9	0.126
Total Sleep time (min)	362.6±58.9	345.1±62.1	0.035
Sleep efficiency (min)	83.9±11.1	82.5±12.5	0.412
Sleep latency (min)	13.2±13.6	12.2±18.1	0.660

Data are expressed as n (%) or mean ± standard deviation. OSA, obstructive sleep apnea; SaO_2_, oxygen saturation.

Among the OSA group, 90 (28.4%) had mild OSA, 77 (24.4%) had moderate OSA, and 149 (47.2%) had severe OSA. The percentages of male patients and those with a smoking history were significantly higher in patients with OSA than in those without OSA. Patients with OSA had a significantly higher age, BMI, WC, and NC than patients without OSA. A higher Epworth sleepiness score was associated with the OSA group.

Women with OSA were older (56.1±10 years vs. 47.3±12.9 years for women and men, respectively; p<0.001) and less obese (BMI, 25.8±4.9 vs. 27.1±3.5 for women and men, respectively; p = 0.036). Significant differences existed in mean NC (34.5±3.5 vs. 39.2±2.8 for women and men, respectively; p<0.001) and WC (89.7±12.2 vs. 96.0±8.9 for women and men, respectively; p<0.001). Despite being younger, the severity of OSA was significantly higher in men compared to women. The mean AHI in men was 38.9±26.1 and 25.4±23.9 in women (Table 2).

**Table 2:** Gender difference in anthropometric and polysomnographic measures of OSA subjects.

	Men (n = 242)	Women (n = 74)	*P value*
Age (years)	47.3±12.9	56.1±10.2	<0.001
Body mass index (kg/m^2^)	27.1±3.5	25.8±4.9	0.036
Waist circumference (cm)	96.0±8.9	89.7±12.2	<0.001
Neck circumference (cm)	39.2±2.8	34.5±3.4	<0.001
Epworth sleepiness scale	9.5±4.2	9.0±4.2	0.354
Mean SaO_2_ (%)	91.9±3.8	92.8±4.5	0.086
Lowest SaO_2_ (%)	78.9±8.2	80.1±9.3	0.299
Apnea-hypopnea index (/h)	38.9±26.1	25.4±23.9	<0.001

Data are expressed as mean ± standard deviation. OSA, obstructive sleep apnea; SaO_2_, oxygen saturation.

**Figure 1:**
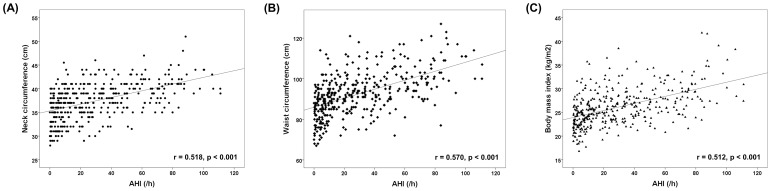
Correlations between the anthropometric indices and the apnea-hypopnea index (AHI).

All anthropometric indices (NC: r = 0.518, p<0.001; WC: r = 0.570, p<0.001; BMI: r = 0.512, p<0.001) were significantly correlated with AHI (Figure 1). After adjusting for age, sex, alcohol consumption, and smoking, a logistic regression model that was used to evaluate the significance of the individual anthropometric indices for the presence of OSA showed that NC [Odd ratio (OR), 1.414; 95% confidence interval (CI), 1.24–1.62; p<0.001)], WC (OR, 1.114; 95% CI, 1.07–1.16; p<0.001), and BMI (OR, 1.364; 95% CI, 1.22–1.53; p<0.001) were significantly associated with the presence of OSA. A linear regression model that was used to evaluate the significance of the individual anthropometric indices for the severity of OSA showed that NC (β = 3.748, p<0.001), WC (β = 1.272, p<0.001), and BMI (β = 3.082, p<0.001) were significantly associated with AHI (Table 3).

**Table 3:** A logistic and linear regression analysis to identify the significance of anthropometric indexes for the presence and the severity of OSA.

	Presence of OSA		AHI		
	OR (95% CI)	*P value*	*β*	SE	*P value*
Body mass index	1.364 (1.22–1.53)	<0.001	3.082	0.304	<0.001
Neck circumference	1.414 (1.24–1.62)	<0.001	3.748	0.412	<0.001
Waist circumference	1.114 (1.07–1.16)	<0.001	1.272	0.116	<0.001

Adjusted for age, sex, alcohol consumption, and smoking, OSA, Obstructive sleep apnea; AHI, apnea-hypopnea index, OR, odds ratio; β, standardized regression coefficient; SE, standard error; CI, confidence interval.

**Figure 2:**
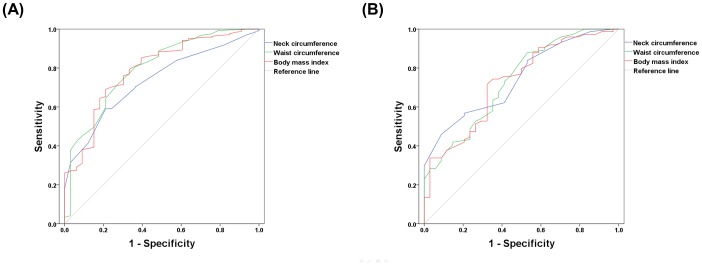
Receiver Operating Characteristic (ROC) analysis to determine the optimal cut-off values of the individual anthropometric indices for predicting obstructive sleep apnea (OSA) in (A) females and in (B) males. The values 34.5 cm for neck circumference (NC, sensitivity, 50%; specificity, 89%), 76.5 cm for waist circumference (WC, sensitivity, 88%; specificity, 54%), and 23.05 kg/m^2^ for body mass index (BMI, sensitivity, 72%; specificity, 68%) were found optimal for females; the values of 38.75 cm for NC (sensitivity, 60%; specificity, 79%), 88.5 cm for WC (sensitivity, 81%; specificity, 64%), and 24.95 kg/m^2^ for BMI (sensitivity, 70%; specificity, 79%) were found optimal for males.

In the ROC analysis that was used to determine the optimal cut-off values of the individual anthropometric indices for predicting OSA, the values of 34.5 cm for NC (sensitivity, 50%; specificity, 89%), 76.5 cm for WC (sensitivity, 88%; specificity, 54%), and 23.05 kg/m^2^ for BMI (sensitivity, 58%; specificity, 88%) were optimal for females, and the values of 38.75 cm for NC (sensitivity, 60%; specificity, 79%), 88.5 cm for WC (sensitivity, 81%; specificity, 64%), and 24.95 kg/m^2^ for BMI (sensitivity, 70%; specificity, 79%) were optimal for males (Figure 2, Table 4). When we assessed the area under the ROC curves with paired comparisons for NC, WC, and BMI, there were no statistically significant differences between the anthropometric indices in both genders.

**Table 4:** Cut-off values for neck circumference, waist circumference, body mass index as predictor of OSA in male and female.

	Neck circumference		Waist circumference		Body mass index	
	Male	Female	Male	Female	Male	Female
Area under the ROC curve (95% CI)	0.73 (0.65–0.81)	0.74 (0.65–0.84)	0.80 (0.71–0.88)	0.73 (0.63–0.83)	0.79 (0.71–0.87)	0.73 (0.63–0.83)
Cut-off value	38.75 cm	34.5 cm	88.5 cm	76.5 cm	24.95 kg/m^2^	23.05 kg/m^2^
Sensitivity	60%	50%	81%	88%	70%	72%
Specificity	79%	89%	64%	54%	79%	68%

OSA, Obstructive sleep apnea; ROC, receiver operating characteristics; CI, confidence interval.

## Discussion

Our study analyzed various anthropometric measures and their associations with the presence, as well as the severity, of OSA in the Korean adult population. To the best of our knowledge, this is the first study to determine the cut-off values for predicting the risk of OSA in anthropometric indices including, NC, WC, and BMI, in an Asian population.

In this study, patients with OSA had statistically significantly higher BMI, WC, and NC values compared to the patients without OSA. All of the anthropometric indices (NC, WC, and BMI) were significantly correlated with the severity of OSA. These results were similar to those of previous studies. Young et al. have reported that the association between obesity and SDB is substantial, with high BMI contributing to moderate to severe SDB in 58% of the affected persons [16]. Yamagishi and colleagues have examined the prevalence of OSA among Hispanic and white Americans and in people of Japanese origin [17]. In spite of the race/ethnic difference, BMI and SDB were strongly associated in all races, and the prevalence of OSA best corresponded with differences in BMI. A recent similar preliminary study of 119 subjects from Korea evaluated the influential clinical and anthropometric characteristics that affected AHI in suspected OSA patients [18]. In the literature, BMI is the most influential factor that affects AHI, and weight, NC, WC, and hip circumference are significant correlated with AHI. Davidson et al. have shown that WC correlates with most significantly with AHI for both men and women in a cohort of 414 patients [10].

In our study, a logistic regression analysis showed that NC was a more potent predictor for the presence of OSA compared to WC and BMI. A linear regression analysis also revealed that NC was more predictive of the severity of OSA compared to the other anthropometric indices.

Clinically, NC has been reported to be a useful predictor of OSA. Simpson and colleagues have evaluated the relationship between the severity of OSA and measures of regional obesity in 96 adults in a prospective case series observational study [7]. They found that NC was associated with both genders. The percentage of fat in the neck region and BMI together explained 33% of the variance in AHI in women.

Obesity, especially central obesity, is a major risk factor for OSA [2]. Many simple anthropometric indices, including WC, BMI, and the waist-to-hip ratio, are widely used as markers of obesity or central obesity. The criteria of obesity that use these anthropometric indices are applied differently according to sex and ethnicity because body-fat distribution and obesity severity depend on a complex interaction of genetic and environmental influences [19]–[21]. Recently, NC has been identified as an index of central obesity and a potential predictor of OSA. Onat et al. [5] have reported that NC contributes to metabolic syndrome likelihood beyond WC and the components of metabolic syndrome. In 92 short-sleeping obese women, Cizza et al. have reported that a NC of ≥38 cm had a sensitivity of 54% and 58% and a specificity of 70% and 79% in predicting the presence of metabolic syndrome and OSAS, respectively [6]. Soylu et al. have reported that NC has a greater value than WC regarding an association with OSA in Turkish adults [11]. They suggested that the optimal cut-off values of NC for predicting OSA were 35.5 cm in females and 39 cm in males. Zhou et al. have reported that NC independently contributes to predict cardio-metabolic risks beyond the classical anthropometric indices and that NC values of 33 cm for females and 37 cm for males are the optimal cut-off values for metabolic syndrome in adults from China [21]. Interestingly, these studies have suggested similar cut-off values of NC for predicting OSA despite the examination of subjects of different ethnicities. In addition, the sex differences in the optimal cut-off values of NC, which was approximately 4 cm, were also like, and this was similar to the results of these studies. Martins and colleagues from Brazil have shown a similar trend in the literature [22]. In this study, cut-off value that was determined for NC for OSA risk was 40 cm for males and 36 cm for females. Another study by Davidson et al. from the United States has reported that 43 cm for males and 38 cm for females determined the presence of OSA [10]. In the present study, all of the anthropometric indices, including BMI, NC, and WC, were significantly associated with the presence of OSA, and the optimal cut-off values of NC for predicting OSA were 34.5 cm in females and 38.75 cm in males. In addition, NC was more closely associated with AHI than other anthropometric indices after adjusting for the confounding factors in this study. In light of these results, NC might be a more useful independent predictor for OSA that is less influenced by ethnicity differences than the other anthropometric indices.

The cut-off value for WC that has been demonstrated as a risk factor of OSA was over 94 cm in males and more 80 cm in females in a previous study [22]. In a study by Soylu et al., the cut-off value of WC was found to be 105 cm in males and 101 cm in females in a Turkish population [11]. In our study, a WC value over 88.5 cm for males and over 76.5 cm for females determined the risk of OSA. Our cut-off values were lower than the values reported in the previous literature. Generally, abdominal obesity is defined as a WC of 90 cm or more in men or 80 cm or more in women in the Asian population and 94 cm or more in men or 80 cm or more in women in Middle East and Caucasian [23]. Despite suffering from a similar severity of OSA, Asian patients are less obese compared to Caucasian patients, suggesting that ethnicity may differentially contribute to OSA [24]. Although there are differences in the abdominal obesity criteria between races and the cut-off values for WC in Western countries are higher than those in Asian countries, our cut-off points were lower than the Asian criteria. In other words, Korean patients tended to have OSA at WC values that are considered normal by the Asian criteria of abdominal obesity. These results suggested that abdominal obesity in the Korean population may differentially influence the development of OSA compared to other ethnicities, and that the impact of obesity on OSA might be more pronounced in the Korean population.

Martin et al. have reported that a BMI>30 kg/m^2^ in both genders is associated with the development of OSA [22]. Soylu et al. have shown that BMI values over 27.77 kg/m^2^ in females and over 28.93 kg/m^2^ in males increase the risk of OSA in a Turkish population [11]. The cut-off value of BMI for obesity in the Caucasian population is 30 kg/m^2^ but some Asian populations have redefined obesity at a lower BMI of 25 kg/m^2^ [25]. For many Asian populations, the current World Health Organization (WHO) BMI cut-off values have been identified as 23 kg/m^2^ or higher for an increased risk for obesity, 25 kg/m^2^ or higher for being overweight, and 27.5 kg/m^2^ or higher for a high risk for obesity [26]. In the present study, the cut-off value for BMI as an OSA determinant was over 23.05 kg/m^2^ in females and over 24.95 kg/m^2^ in males in a Korean population. The cut-off values that we found were lower than those previously reported in the literature. Asians generally have a higher percentage of body fat than Caucasian of the same age, sex, and BMI [26]. For this reason, a proportion of the Asian population with risk for OSA might have BMIs below the existing WHO BMI cut-off value of 25 kg/m^2^. In addition, there are few studies on the various phenotypic features that may contribute differently to the development of OSA. Lee et al. have evaluated the differences in craniofacial structure and obesity in 150 adults with OSA (74 Caucasians and 76 Chinese) [24]. In this interethnic study, Chinese patients with the same degree of obesity as Caucasians had more severe OSA and more craniofacial bony restriction. When the OSA severity was similar, the Caucasian patients had a higher BMI and a larger NC, whereas the Chinese patients exhibited more craniofacial bony restriction. Therefore, genetics or craniofacial structural factors might contribute to the risk of OSA beyond obesity.

In the current study, we found that there was a marked gender difference in the prevalence and severity of OSA. Within the study sample, the males, on average, were younger and heavier, and they had thicker necks and a higher AHI than the females. This finding was consistent with those of previous studies. Subramanian and colleagues have reported that women with OSA have a lower respiratory disturbance index compared to males, despite their higher age, and the severity of OSA was significantly higher in males [27]. Another study by Simpson et al. has shown that there were substantial sex-based differences in the association between fat distribution and the severity of OSA [7]. The mechanisms that potentially explain the difference between men and women with respect to the prevalence and severity of OSA include the following: significant variation in body fat distribution, upper airway collapsibility, hormonal status, and control of ventilation [28], [29], [30]. In particular, sex hormone status is a well-known risk factor for OSA in women [31]. Although our data did not include the menopausal status of the females, the mean age of the women in this study (56.1±10 years) was very close to postmenopausal age.

The present study had some limitations. First, because our study was a cross-sectional analysis, there was the possibility that confounding factors that were unaccounted for may have resulted in biased results. Second, because of the usual male predominance in referrals to sleep clinics, there was an unbalanced gender distribution. Third, because only high-risk subjects with suspected OSA were included in this study, the results should be applied with caution to the general population. Finally, we did not objectively assess the amount and distribution of adiposity in the study population. However, our results suggested that the analysis of anthropometric indices might provide useful information for a presumptive diagnosis for OSA. Moreover, the chosen cut-off values can be easily applied in the clinical field.

In conclusion, in our study group, all anthropometric indices including, BMI, NC, and WC, were significantly associated with the presence and severity of OSA in a Korean population. NC significantly correlated with OSA and might be of better use than other indices when describing a patient with OSA. In addition, this study demonstrated the cut-off values of BMI, WC, NC that increase the OSA risk.
